# Solvent assisted size effect on AuNPs and significant inhibition on K562 cells†

**DOI:** 10.1039/c9ra05484g

**Published:** 2019-10-22

**Authors:** Chander Amgoth, Avinash Singh, Rompivalasa Santhosh, Sujata Yumnam, Priyanka Mangla, Rajendra Karthik, Tang Guping, Murali Banavoth

**Affiliations:** Department of Science and Humanities, MLR Institute of Technology Hyderabad-500043 TS India; Department of Electronics and Communication Engineering, MLR Institute of Technology Hyderabad-500043 TS India; School of Chemistry, Zhejiang University Hangzhou-310028 China; School of Chemistry, University of Hyderabad Hyderabad-500046 TS India

## Abstract

Herein, the synthesis and characterization of ideal size (∼10 and 40 nm, in diameter) AuNPs (gold nanoparticles) were reported. Two different organic solvents such as DMF (dimethyl formamide) and NMPL (*N*-methyl-2-pyrrolidone) were used to synthesize AuNPs along with agents reducing agents such as NaBH_4_ (sodium borohydrate) and Na_3_C_6_H_5_O_7_ (sodium citrate). The combination of [(HAuCl_4_)–(DMF)–(NaBH_4_)] gives AuNPs with an avg. size of 10.2 nm. Similarly, the combination of [(HAuCl_4_)–(NMPL)–(Na_3_C_6_H_5_O_7_)] gives AuNPs with an avg. size of 40.4 nm. The morphology of these nanoscale AuNPs has been characterized through TEM and HRTEM imaging followed by SAED for lattice parameters such as *d*-spacing value (2.6 Å/0.26 nm) of crystalline metal (Au) nanoparticles. Further, these unique and ideal nanoscale AuNPs were used to evaluate the potential working efficacy by using *in vitro* cell based studies on K562 (leukaemia) blood cancer cells. From the MTT assay results around 88% cell inhibition was measured for ∼10 nm sized AuNPs. The treated cells were stained with different fluorescent dyes such as FITC, DAPI, Rho-6G and their ruptured morphology has been reported in the respective sections. These types of ideal sized metal (Au) nanoparticles are recommended for various theranostics such as to cure breast, colon, lung and liver cancers.

## Abstract 2

Herein, the synthesis and characterization of ideal size (∼10 and 40 nm, in diameter) AuNPs (gold nanoparticles) were reported.

## Graphical abstract

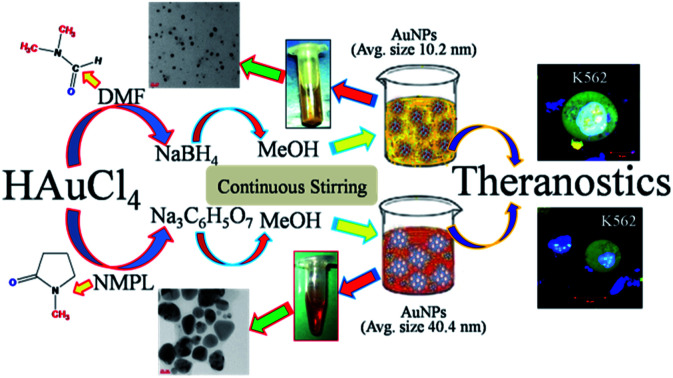

## 1. Introduction

There is the possibility to stabilize smaller sized nanoparticles through the increased polarity of solvents.^1^ Solvents stabilize the system through charge stabilization and capping approaches. As per the classical homogeneous nucleation theory, the homogeneous nucleation leads to growth of the particles spontaneously with the solvent effect.^1,2^ As per the supersaturation concept, the ionic product divided by solubility product helps in the homogeneous nucleation of AuNPs. The decrease in the solubility of an ionic material with non-polar solvent will show an adverse affect on the size of AuNPs followed by the heterogeneous nucleation. And this can be avoided by choosing the appropriate polar solvent for homogeneous, consistent nucleation for metal (Au) nanoparticles.^2,3^ With this chemical precipitation method, there is a high chance to get homogeneous nuclei followed by consistent sized particles and this happens because of the solvent and its concentration. Such small AuNPs and the solvent effect has been applied for novel metal nanoparticles with various applications.^1–4^

The oxalate based solvents lead to the single crystal formation for smaller nanoparticles but aggregation and agglomeration phenomena can rely on the chemical precipitation and show the adverse effect to enhance the particle sizes.^5^ The RSS (relative supersaturation) concept gives some important information to control the nucleation and crystal growth followed by the chemical precipitation methods.^5,6^ The different concentrations of reagents and solvents do show the adverse affect followed by the capping of metal (Au) nanoparticles. Sometimes atmospheric conditions such as temperature, pH, stirring methods also show the effect on the size of particles.^7^ The redox (oxidation and reduction) reaction of HAuCl_4_ (tetrachloroauric acid) with MoS_2_ (molybdenum disulfide) leads to the formation of [(AuNPs)–(MoS_2_)] nanocomposite.^5–8^ The organic solvent DMF (dimethyl formamide) shows higher rate of reduction on gold materials such as Au^3+^ (gold III) which gives smaller sized particles.^1,8,9^ However, organic solvent NMPL (*N*-methyl-2-pyrrolidone) has the lower reduction rate on metal/gold nanoparticles and it leads to the larger sized particles.^10,11^ From the DMF and NMPL organic solvents it is evidenced that there is a chance to synthesize the gold nanoparticles in the size range of 8 to 40 nm (in diameter). With these organic solvents the stepwise increase in the size of metal (Au) nanoparticles can be achieved. Gold nanoparticles (AuNPs) have been under wide range of utilization since last couple of decade, especially industrial and pharmaceutical/biomedical fields.^1,9–12^

Recently, these ideal sized metal (Au) nanoparticles have been used in various technological and biomedical applications such as organic photovoltaic,^9^ drug delivery systems (DDSs),^10^ sensory probes,^11,12^ pharmaceutical and therapeutic applications, catalysis and electronic conductors.^11–14^ The size, shape and morphology/geometry of gold nanoparticles are changeable by tuning their physical and chemical properties.^1–5,15^ These polar solvents propagate the interactions between the solvent molecules and metal particles to reduce the size and change the surface chemistry of nanoparticles through the aggregation and this aggregation can be avoided through the sonication methods. The monodispersed AuNPs with size range of 8–40 nm are most suitable for killing and to inhibit the proliferation of cancer cells.^16^ The concept surface plasmon resonance can help to kill the cancer cells predominantly through the dosage based localization inside the cells.^16,17^ These ideal sized AuNPs are most suitable tools to penetrate the cell membrane and to show the killing and inhibition activity significantly. The concept surface plasmon resonance depends on the size of metal (Au) nanoparticles and it can be applied for various theranostics. Interestingly, these gold nanoparticles (AuNPs) have been used for extensive studies based on their colloidal stability, surface chemistry, capping nature, surface tunability based on its chemical and physical properties. All these properties will do change from bulk to nanoscale sizes.^18^ Particularly, the AuNPs with the size range of 10–40 nm are of great interest due to the interface science between bulk to nanoscale reduction followed by the molecular regime, and interactions between solvent to metal (Au) nanoparticles. Similarly, these synthesized small sized (10–40 nm) AuNPs are of great interest because of their catalytic properties followed by the easy penetration and incorporation inside the cancer cells and other theranostics.^15–19^

Furthermore, the recent nanotechnological advances in the biomedical field has shown potential working efficacy of these 10–40 nm sized AuNPs can be utilized as contrast agents for blood pool assay and CT (computed tomography) scanning such as tumour computed tomography imaging *etc.* These nanosized AuNPs are ideal and unique in their activities to enhance the image contrast while CT scanning followed by the *in vivo* animal model imaging. These AuNPs not only used in CT scanning, they can be used in PDT (photodynamic theory) for various treatments and surgeries based on their ideal particle size suitability and changeability. The ideal sized (10–40 nm) AuNPs are recommended for hyperthermia therapy to kill the cancer cells through the rapid heating of gold nanoparticles and interactions with the cancer cells lead to rupture the cells.^20^ However, these AuNPs can be coated or loaded with the nanomedicines and anticancer drugs and referred as nanoformulations as therapeutic agents for drug delivery systems (DDSs) to treat and cure various diseases and ailments. The synthesis and characterization of nanosized gold particles (AuNPs) are used in various fields such as therapeutic agents, sensory applications, nanobiotechnology, catalysis, calorimetric applications, MRI and CT scanning, to know the vibrational and chemical bonding energies and strengths, detection of proteins, genetic material, pollutants and other industrial and pharmaceutical applications.^17–22^ These nanoscale AuNPs can be used for many diagnostic methods such as detection of biomarkers, heart diseases, lung, liver, prostate, colon cancers and other infectious diseases. The AuNPs (gold nanoparticle) surface can be used as selective oxidation followed by the surface modifications for various fuel cell and automotive applications.^21,23,24,29^

## 2. Materials and methods

### 2.1 Materials

All the chemicals required for the synthesis of nanoscale AuNPs has been purchased with high purity and used without further purification. The HAuCl_4_ (tetrachloroauric acid, ∼98.9%, Sigma Aldrich), DMF (dimethyl formamide, 97.6%, Sigma Aldrich), NaBH_4_ (sodium borohydrate, >97.8%, SDFCL), NMPL (*N*-methyl-2-pyrrolidone, >98%, Sigma Aldrich), Na_3_C_6_H_5_O_7_ (sodium citrate, >98%, SDFCL), IPA (isopropanol, >98%, SDFCL), methanol (>96%, SDFCL), acetone extra pure (>96%, sigma Aldrich), carbon coated copper grids with 200 mesh size (Ted Pella Inc), MTT (methyl thiazol tetrazolium, >99.9%, Sigma Aldrich), magnetic bead, magnetic stirrer cum hot plate, sample viols, micropipettes and centrifuge tubes. Apart from these chemicals, various other glassware, consumables such as beakers, stirrer, magnetic bead, hot plate, goggles, gloves *etc.*

### 2.2 Synthesis of AuNPs with an avg. size of 10 nm

The chemical compound HAuCl_4_ (tetrachloroaurate/auric acid) of ∼2.5 g was taken in a 250 mL RB flask (round bottom flask). This has been added with ∼60 mL of organic solvent DMF (dimethyl formamide) and allowed to stir for 12 h at 50 °C. This is again added with the 0.5 g of NaBH_4_ (sodium borohydrate) strong reducing agent and allowed to stir for 2 h at 50 °C. Furthermore, to that mixture methanol (MeOH) ∼25 mL has been added and allowed to stir for 2 h at 50 °C.^1,11,17,25^ Finally, the sodium metal from reducing agent NaBH_4_ was degraded with the methanol solvent. The end product of reaction was yellowish in colour and this solution was used to separate the metal (Au) nanoparticles through the high speed centrifugation at 10 000 RPM for 10 minutes. The final gold nanoparticles were dispersed in IPA (isopropanol) solution and used to prepare the samples for TEM, HRTEM and SAED characterizations. The physical appearance of final gold sample has been reported in the inset of the Fig. 6a and its respective dispersion of gold nanoparticles with TEM micrographic imaging. The yield of the final synthesized nanoscale AuNPs is calculated as 1.7/2.5 g (w/w%) which is equal to ∼68%.

### 2.3 Synthesis of AuNPs with an avg. size of 40 nm

The chemical compound HAuCl_4_ (tetrachloroaurate/auric acid) of ∼2.5 g was taken in a 250 mL RB flask (round bottom flask). This has been added with ∼60 mL of organic solvent NMPL (*N*-methyl-2-pyrrolidone) and allowed to stir for 12 h at 50 °C. It is again added with the 0.5 g of Na_3_C_6_H_5_O_7_ (sodium citrate, >98%, SDFCL), strong reducing agent and allowed to stir for 2 h at 50 °C. Furthermore, to that mixture ∼25 mL of methanol (MeOH) has been added and allowed to stir for 2 h at 50 °C. Finally, the sodium metal from reducing agent Na_3_C_6_H_5_O_7_ was degraded with the methanol solvent. The end product of reaction was dark red in colour and this solution was used to separate the metal (Au) nanoparticles through the high speed centrifugation at 10 000 RPM for 10 minutes.^1,11,17,26–29^ The final AuNPs were dispersed in IPA (isopropanol) solution and used to prepare the samples for TEM, HRTEM and SAED characterizations. The physical appearance of final gold sample has been reported in the inset of the Fig. 6b and its respective dispersion of gold nanoparticles with TEM micrographic imaging. The yield of the final synthesized nanoscale AuNPs is calculated as 2.0/2.5 g (w/w%) which is equal to ∼80%.

### 2.4 Sample preparation for TEM imaging

The synthesized AuNPs of 0.2 g was dispersed in 3 mL of IPA (isopropanol) solution and used for the sample preparation for TEM, HRTEM and SAED characterizations. The dispersion followed by the ultra sonication of AuNPs with the help of isopropanol solvent. Then, the ∼20 μL of well dispersed solution of AuNPs was drop casted on the surface of the carbon coated copper grids with the help of micropipette and grids were allowed to dry for over knight. The well dried samples were used for TEM imaging at 200 kV accelerating voltage.^1,11,17,19,30^ These crystalline metal (Au) nanoparticles does not show any charge effect at such higher accelerating voltages because of their high melting point and its crystalline nature.

## 3. Results and discussions

### 3.1 Morphology of AuNPs

**Fig. 1:**
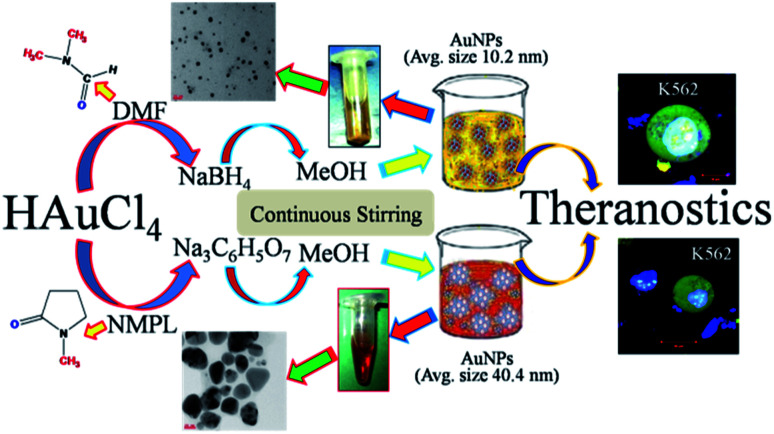
Schematic illustrate the steps involved in the synthesis of nanoscale AuNPs followed by the required solvents, reducing agents and source of gold nanoparticles and experimental procedures.

The Fig. 1 schematic illustrate the steps involved in the synthesis of nanoscale AuNPs^31^ followed by the required solvents, reducing agents and source of gold nanoparticles and experimental procedures. The synthesis of ideal sized nanoscale gold nanoparticles (AuNPs) with changeable size, shape, and morphology and surface properties is a unique and novel approach for the scientific research community.^28–32^ The spherical shaped gold nanoparticles are scientifically useful for the many industrial and pharmaceutical applications. This can be achieved through the selection of solvents, reducing agents, concentration, temperature, pH of the system, sonication methods *etc.* The organic solvents dimethyl formamide (DMF) and *N*-methyl-2-pyrrolidone (NMPL) has the role in reduction of the size of the gold nanoparticles along with the reducing agents such as sodium citrate (Na_3_C_6_H_5_O_7_) and sodium borohydride (NaBH_4_). The solvent methanol (MeOH) has the property to degrade and decompose the sodium (Na) metal to get the ideal nanoscale particles. The Fig. S1 and S2† correspond to TEM micrographs of AuNPs synthesized from DMF and NaBH_4_. These images were acquired from lower to higher magnification and shows ideal nanosized gold particles.

**Fig. 2:**
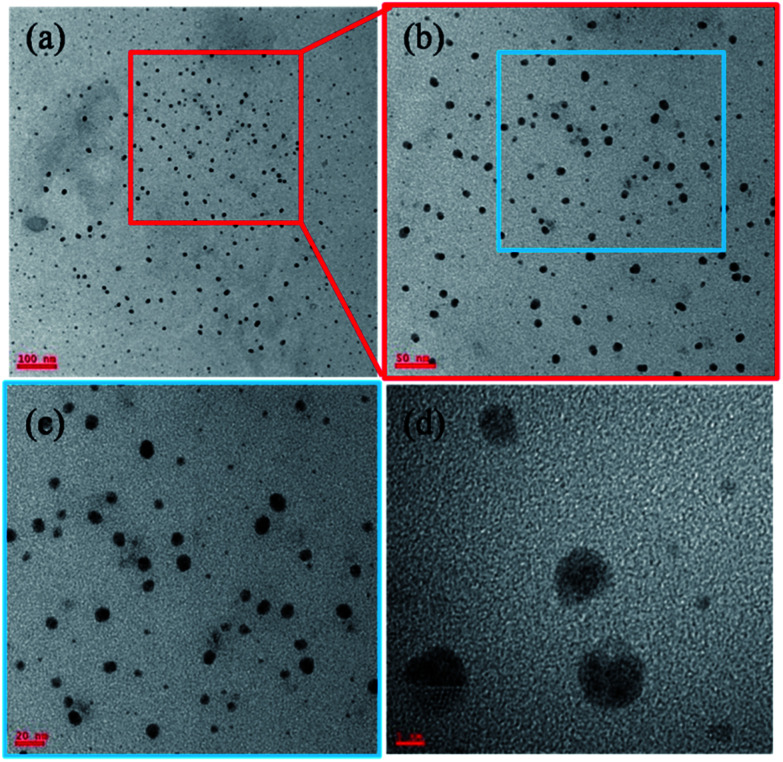
TEM micrographs of smaller (avg. particle size is 10.4 nm), gold nanoparticles (AuNPs). The images (a–d) are acquired from lower to higher magnification.

The Fig. 2a–d corresponds to the TEM micrographs acquired from lower to higher magnification such as scale bar of 100, 50, 20 and 5 nm respectively. As the magnification increases, the depth of focus (DOF) decreases and it leads to reduce the scanning area with that less number of nanoparticles can be seen in higher magnified images and the Fig. 2d has very few nanoparticle at higher magnification. These are smaller gold nanoparticles with an average particle size of 10.4 nm.^31–33^

**Fig. 3:**
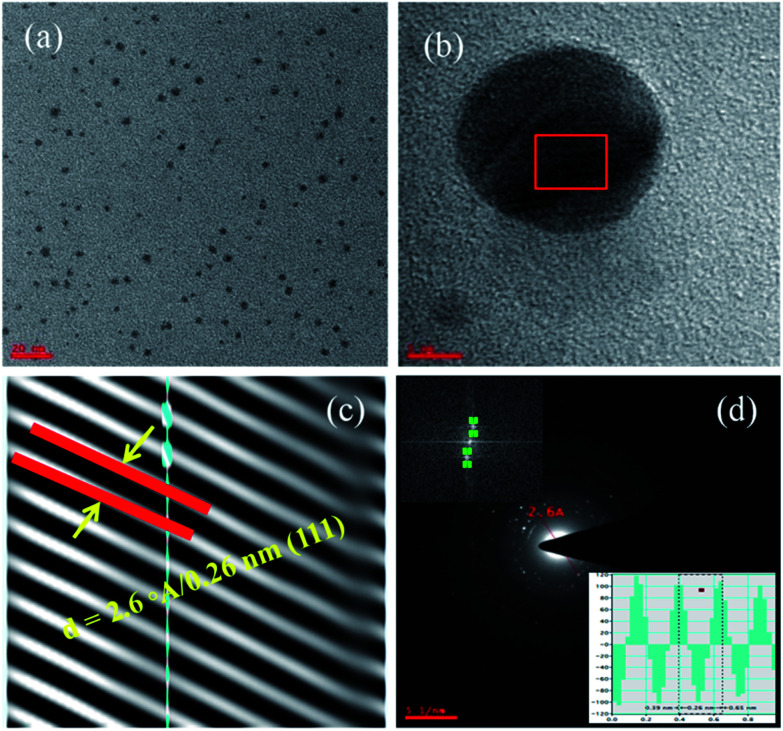
TEM micrographs (a and b) for AuNPs are acquired from lower to higher magnifications. The image (c) corresponds to AuNPs lattice fringes which corroborates the *d*-spacing value of 0.26 nm and image (d) corresponds to SAED for AuNPs and its IFFT (inset) evidences the *d*-spacing value.

These ideal nanosized gold nanoparticles (AuNPs) were synthesized by using the DMF organic solvent along with strong reducing agent sodium borohydrate (detailed synthesis mentioned in the Section 2.2 of materials and methods). The HAuCl_4_ has been used as source precursor materials for gold nanoparticles and reaction was performed in the methanol solvent at 50 °C for several hours. From the Fig. 2a–d TEM micrographs, the monodispersed and well distributed AuNPs are observed. The surface chemistry of AuNPs depends on the solvent and concentration of solvent followed by the formation of capping agents on the surface of the nanoparticles. In this case, AuNPs size is almost consistent with monodispersion of particles throughout the sample or TEM grid surface.^29–34^ These AuNPs are spherical shaped with insistent size and dispersion throughout the surface and it rely on the reducing agents used during the synthesis process. The harsh reducing agent encourages the heterogeneous nucleation and it lead to give highly inconsistent size, shape and irregular dispersion of nanoparticles. The sonication methods, temperature of the sonication bath, sonication time will also affect the consistency in getting the uniform sized nanoparticles. The Fig. 3a and b corresponds to the AuNPs captured from lower to higher magnification. The Fig. 3a has been acquired at lower magnification and it shows large number of AuNPs.

### 3.2 Interplanar distance and solvent effect on AuNPs

The Fig. 3b acquired at higher magnification and only one nanoparticle has been seen at such higher resolution. It represents the lattice fringes and this image has been used to calculate the lattice parameters such as *d*-spacing (Fig. 3c). To get these *d*-spacing values, the raw data has been imported to Gatan software and used bright spot method to analyze the lattice fringes (inter-planar distances) as reported in the Fig. 3c and it gives 2.6 Å/0.26 nm which is highlighted in the figure. This has been evidenced by the SAED (selected area electron diffraction) in the Fig. 3d and this *d*-spacing value has been measured through the two opposite bright spots of the same circle.^1,11,17,19,30–35^

Further, this *d*-spacing (inter-planar distance) value (0.26 nm) has been confirmed by suing the IFFT method (inset in Fig. 3d) and its corresponding values. Interestingly, these TEM characterization studies disclose the uniqueness of physical and chemical properties dependency on polarity of solvent as well nature of reducing agents with respect to reaction methods and conditions. The size, shape and morphology followed by the smooth and rough surface of the gold nanoparticles is extensively dependent on the variable parameters such as temperature (*T*), volume (*V*), number of moles (*n*), pressure (*P*) of reaction chamber *etc.* From the TEM micrographs (Fig. 2 and 3), it has been confirmed that the synthesized AuNPs are not capped or covered by any residues which means surface of the particles is smooth with spherical shaped morphology. The most important strategy involved in the synthesis of ideal sized AuNPs is dependent on the selection of organic solvent followed by the Schiffrin–Brust approach. This strategy helps in the design and development of AuNPs with tunable size, shape and morphology. This methodology shows significant effect on the size, shape and morphology through the homogeneous nucleation followed by the monodispersion of nanoparticles and consistent size of particles.

However, the crystalline metal (AuNPs) are stable at 200 kV accelerating voltages while TEM imaging. Our main aim is to design and develop ideal sized AuNPs which are in the size range of ≤50 nm and it is achieved through the modified Schiffrin–Brust approach by using organic solvents and strong reducing agents. The Fig. S3† corresponds to TEM images of AuNPs synthesized by using NMPL organic solvent and sodium citrate (Na_3_C_6_H_5_O_7_) which results in the large sized nanoparticles.^1–6,17–23,35^

**Fig. 4:**
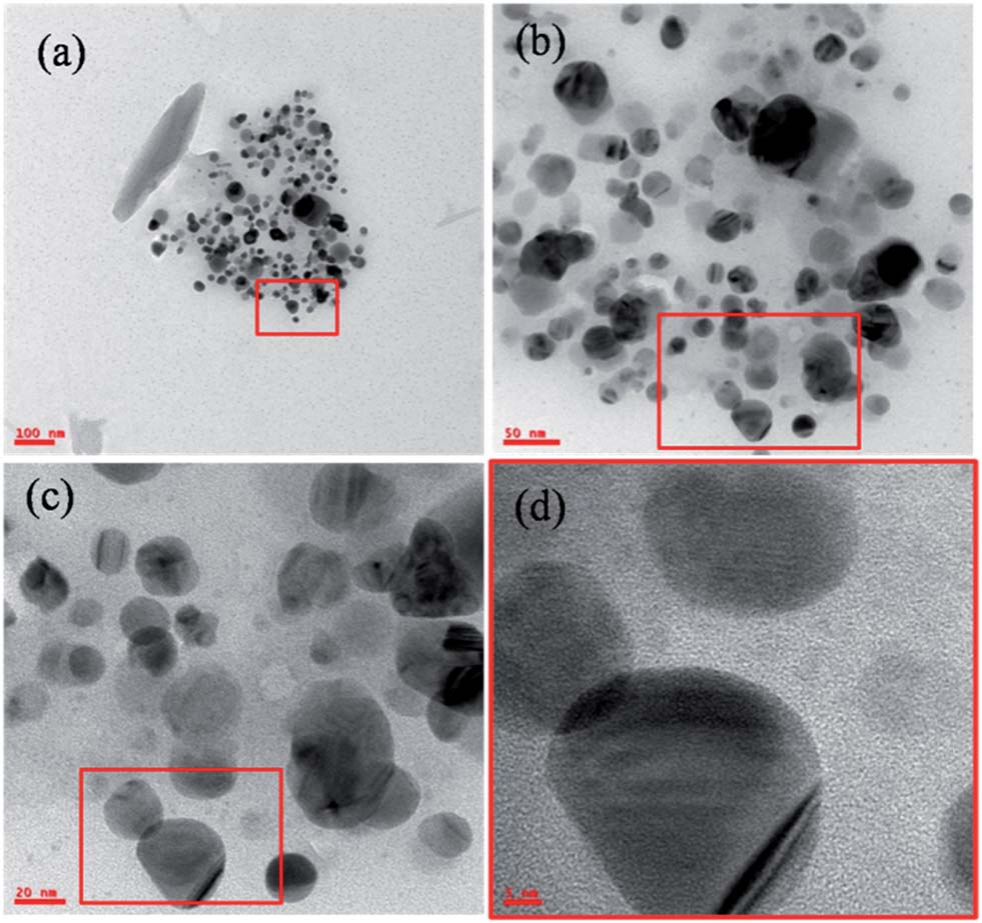
TEM micrographs for larger AuNPs (avg. particle size is 40.2 nm). The images (a–d) are acquired from lower to higher magnification. From the image (d) the lattice fringes can be observed.

The Fig. 4a–d TEM micrographs are acquired from lower to higher magnification and these are synthesized by using the NMPL organic solvent and sodium citrate as strong reducing agent (detailed synthesis mentioned in the Section 2.3 of materials and methods). From these TEM images, the irregular shaped AuNPs are observed with highly inconsistent size of particles. From the Fig. 4b, huge irregularity and inconsistency of AuNPs has been seen. The dispersion and distribution of nanoparticles are interpreted through these TEM images. The highly magnified Fig. 4d corroborates the morphology and lattice fringes of crystalline metal (Au) nanoparticles. The more inconsistency in the size and shape of these AuNPs is due to the heterogeneous nucleation of gold nanoparticles controlled by the NMPL organic solvent and reducing agent (sodium citrate). The hexagonal morphology of gold nanoparticles has been observed through these TEM images and this represents the well segregated nanoparticles without any surface defects. The AuNPs basically show phase changes with organic solvents but here it is quantitatively not showing any adverse affect on the size, shape and morphology of nanoparticles. The increased size in this case is due to the lower affect of organic solvent and reducing agent too. The polarity of the used organic solvent shows adverse affect on the increase and decrease of size of metal (Au) nanoparticle sizes. Here in this case, do note that the sodium citrate (reducing agent) has lower affect and it helps in increase of the particle size from 10–40 nm. However, these AuNPs are unique in their size, shape and morphology which are dependent on the reactions conditions. The samples in the Fig. 1 schematic representation shows light yellow colour for 8–12 nm sized AuNPs and dark red for the 30–40 nm. The Fig. S4† corresponds to EDAX and it represents the elemental composition for synthesized AuNPs. Through the EDAX analysis, the following elements were observed in the sample which results in the presence of oxygen (O) with 55.44 (weight%) and 93.87 (atomic%) is because of impurities present in the sample. The inset table shows the atomic (6.13) and weight (44.56) percentages of Au element. There are high chances to get the elemental composition for Cl (chlorine), W (tungsten), Ag (silver), Nb (niobium) because of impurities present in the sample followed by the external contaminations.

### 3.3 Particle size analysis

**Fig. 5:**
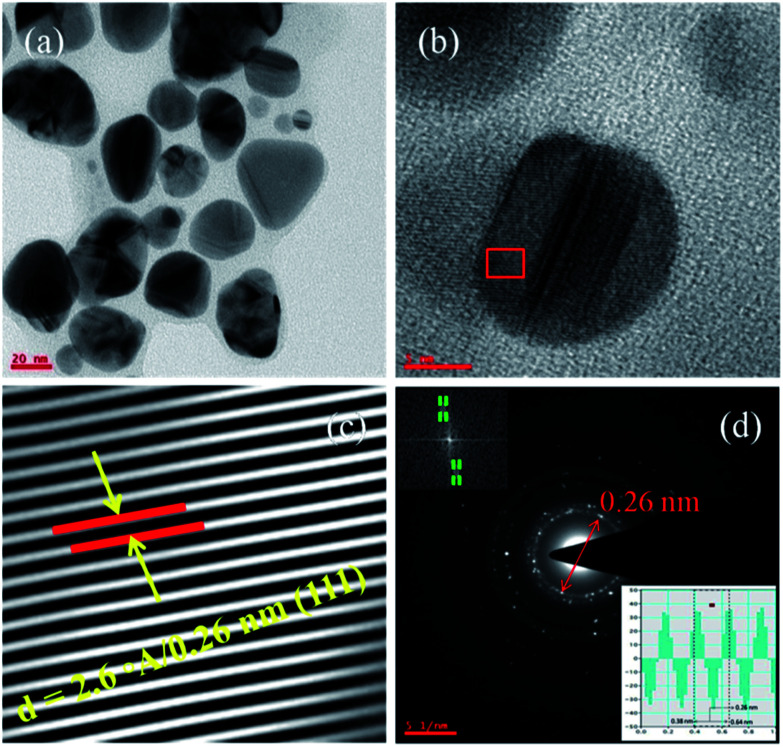
TEM micrographs (a and b) for AuNPs are acquired from lower to higher magnifications. The image (c) corresponds to AuNPs lattice fringes which corroborates the *d*-spacing value of 0.26 nm for larger nanoparticles. The image (d) corresponds to SAED for AuNPs and its IFFT (inset) which evidences the *d*-spacing value.

The crystalline metal (Au) nanoparticles are hexagonal shaped with cubic close packing or FCC (face cantered cubic) crystal lattice structure. From the Fig. 4b and 5b, the HRTEM images inter-planar distances are visible but those cannot be differentiated. In such cases Fig. 4c and 5c for both the (smaller and larger) nanoparticles were analyzed through the DM_3_ data files and enlarged inter-planar distances are reported as 2.6 Å (0.26 nm) corresponds to the (111) crystal plane which is consistent value for both the AuNPs samples. The organic polar environment is the main factor to affect or reduce the AuNPs size along with strong reducing agents.^1–4,19–28,36^ These are showing significant influence on controlling the metal nanoparticle size, shape and morphology through the self-assembly and affinity between metal atoms and polar solvents with the help of reducing agents as size changing agents. The Fig. 5a and b acquired from lower to higher magnification for the larger AuNPs (scale bar 20 to 5 nm) and we can see more number of particles at lower magnification with triangular, hexagonal, spherical and even more irregular shaped gold nanoparticles. From the Fig. 5d, once again the inter-planar distances (2.6 Å/0.26 nm with (111) plane) for the Au metal with FCC crystal system has been evidenced. The inset in Fig. 5d, the IFFT data also evidences the lattice fringes for crystalline metal (Au) nano-system. It also illustrate the (inset) of two bright spots joined for interplaner distances of AuNPs.^27,28,36^

**Fig. 6:**
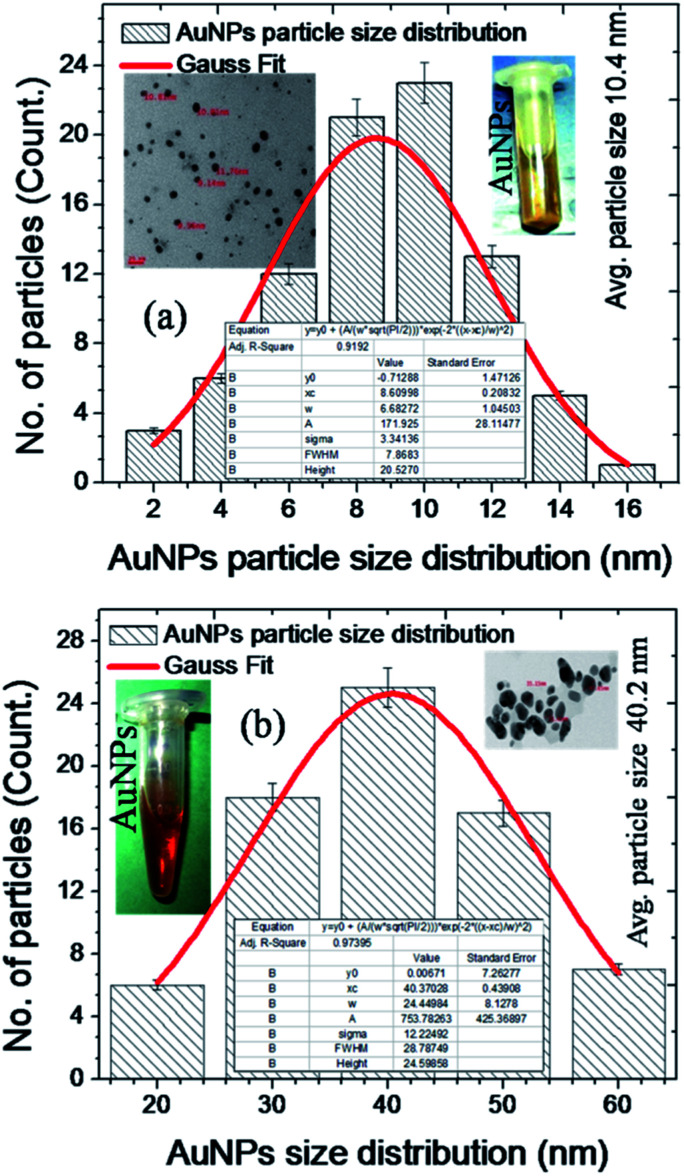
Plot (a and b) shows the AuNPs particles size distribution for smaller (∼10 nm) and larger (∼40 nm) sized nanoparticles.

The Fig. 6a and b corresponds to particle size distribution for the AuNPs synthesized in both the approaches. The Fig. 6a corroborates the particle size distribution for smaller AuNPs whose average particle size is 10.4 nm. The inset sample vial represents the physical appearance (light yellow colour) of these nanoscale gold nanoparticles. Furthermore, inset TEM micrograph illustrates the diameter of particles marked while imaging and size, shape and morphology of synthesized AuNPs with their distribution and dispersion.

The particle size distribution has been calculated through the free hand polygon method which interprets the count of number of particles followed by the size distribution in diameter. The inset table corroborates the parameters involved in the non-linear Gauss curve fit. The regression coefficient (*R*^2^) value for the distribution plot has been obtained as 0.9192 which signifies the best non-linear curve fit after 100 iterations. The FWHM (full width half maxima) value is 7.86 and height value as 20.52 and all other parameter were also incorporated in the respective table. Similarly, the Fig. 6b corroborates the particle size distribution for larger AuNPs. The physical appearance (dark red colour) of these particles can be seen in the sample vial incorporated in the inset of the figure. The inset TEM micrograph illustrates the size, shape and morphology of larger nanoparticles. Some of the particles are marked with their diameter values through the TEM characterizations. The plotted bar graphs represents the average size of AuNPs is 40.2 nm in diameter. The inserted table illustrates the values and parameters for the non-linear Gauss curve fit for larger AuNPs. The *R*-square (*R*^2^) or regression coefficient value for the Gauss fit has been calculated as 0.9739 after 100 iterations and it represents the best curve fit for the large scale sized AuNPs. Furthermore, the FWHM value is calculated as 28.78, sigma value as 12.22 and height value as 24.59 were imbedded in the respective table to easy understanding of all the AuNPs properties and parameters. The AuNPs in the size range of 60–80 nm in diameter can be achieved through the aqueous and non-polar organic solvents. The ideal sized (particle size 8–12 nm) nanoparticles cannot be extracted through the polar organic solvents with strong reducing agents. The AuNPs in the size range of 8–12 are especially attractive for many biomedical and pharmaceutical applications. Herein, these ideal nanoscale AuNPs were synthesized without using any surfactants. As per the literature AuNPs beyond a certain size (>50 nm) are prone to flocculation (coagulation and agglomeration) because of van der Waals's interactions to stabilize the particles. Here nanoscale AuNPs do not show any flocculation and this is happened due to absence of surfactants.^1–8,11,17,27,28,37^

The use of non-polar solvents such as CHCl_3_ (chloroform) and toluene results in the formation of AuNPs with size of >80 nm followed by the flocculation in nanoparticles. The narrow sized gold nanoparticles have good monodispersion with normal solvents like methanol, ethanol, isopropanol, acetone *etc.* These ideal sized AuNPs are stable at room temperature (RT *i.e.* ∼25 °C) and body temperatures (∼37.4 °C). This surfactant free AuNPs are free from flocculation and used for various industrial and pharmaceutical fields due to their indefinite stability at RT and body temperatures. It is worth mentioning that the size or hydrodynamic volume of AuNPs can be increased upon blending with polymers such as PS (poly styrene), PCL (poly caprolactone) *etc.* The Table 1 represents the source materials for gold nanoparticles (HAuCl_4_), organic solvents used for the reactions, reducing agents used during the synthesis of nanoscale AuNPs and average size of final AuNPs obtained through the our modified synthesis methods.^28,32,36,37^

**Table 1:** Illustrate the source of gold nanoparticles, organic solvents used for the synthesis of AuNPs followed by the reducing agents and average size of synthesized AuNPs

S. no	Source of gold	Solvent	Reducing agent	Avg. size of AuNPs
1	HAuCl_4_	DMF (dimethyl formamide)	NaBH_4_	10.4 nm (Fig. 6a)
2	HAuCl_4_	NMPL (*N*-methyl-2-pyrrolidone)	Na_3_C_6_H_5_O_7_	40.2 nm (Fig. 6b)

From the TEM micrographs, it is disclosed that the AuNPs dispersions demonstrate the long-term stability of particles without any capping agents on the surface of the particles helps in easy penetration and insertion inside the infected cells of human body. Further, these ideal sized AuNPs were used to evaluate the working efficacy on K562 (leukaemia) blood cancer cells and results were incorporated in the following sections.^1,28,32,36–38^

**Fig. 7:**
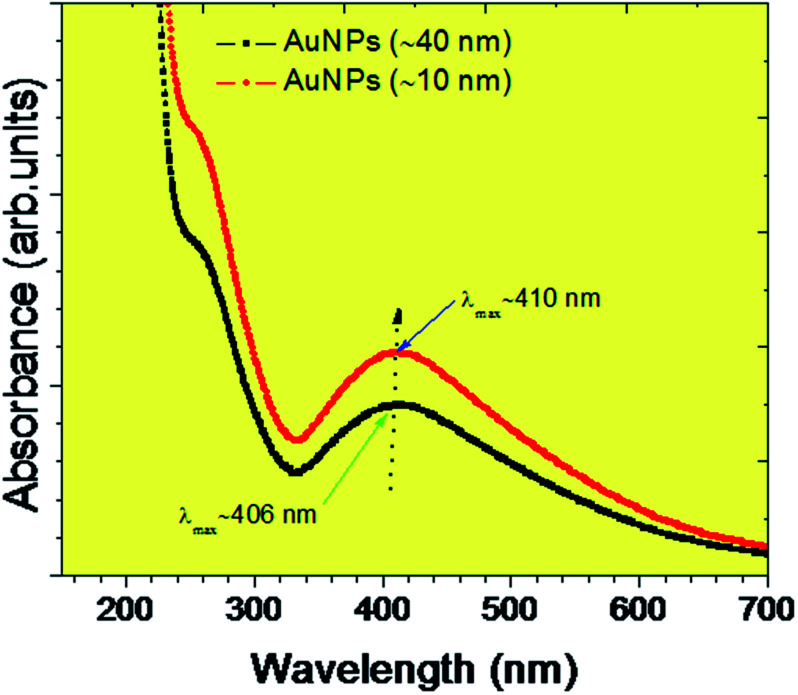
Plot corroborate the UV-visible absorbance of two different sized gold (AuNPs) nanoparticles such as for ∼10 and 40 nm.

The UV-visible absorbance spectrum (Fig. 7) corroborates the absorbance of two different sized gold nanoparticles. The smaller and ideal sized AuNPs of avg. Size 10.4 nm has the sharp peak compare to AuNPs of avg. Size 40.2 nm. The highest and most significant peak for the synthesized AuNPs has been obtained at the wavelength (*λ*_max_) of ∼408 for both sized gold nanoparticles. Around 30 μL of each sample has been used for UV-visible absorption spectrum. The inset in Fig. 7 represents the physical appearance of gold nanoparticle sample. The UV-visible absorbance for AuNPs samples were performed at different concentrations such as AuNPs-I at 30 μL, 60 μL, 90 μL, 120 μL, respectively. Similarly for AuNPs-II sample and spectra for 30 μL of both the AuNPs-I and II has been appended. From the UV-visible spectrum, it has been observed that the peak intensity has been reduced with the increase of size of AuNPs. The shifting in peak position from ∼406 nm to ∼410 nm has been observed for both the 40 and 10 nm sized AuNPs.

### 3.4 IC_50_ on K562 cells and bacterial species

The IC_50_ (minimum inhibitory concentration) of different size AuNPs on leukemia blood cancer cells (K562) has been performed and results were appended in the respective sections. The minimum inhibitory concentrations (MIC/IC_50_) for small (10 nm) sized gold nanoparticles has been obtained to be 0.404 (±0.022) μM. Whereas IC_50_ values for large (40 nm) sized gold nanoparticles has been obtained to be 0.198 (±0.32) μM. From these IC_50_ values it is concluded that small (10 nm) sized AuNPs are potential tools to penetrate the cancer cell (K562) membrane and shows enhanced activity to kill and rupture the cell.

Furthermore, minimum inhibitory concentration (MICs) of two types of gold nanoparticles was evaluated by using different bacterial species such as Gram positive bacteria (*Bacillus subtilis*, *Bacillus cereus*, and *Staphylococcus aureus*) and Gram negative bacteria (*Klebsiella pneumoniae*, *Proteus mirabilis*, and *Escherichia coli*). All the bacterial species were grown and cultured in Muller–Hinton (MH) broth growth medium with humidified atmospheric conditions maintained through the incubation at ∼37 °C over night. The inhibition on both the bacterial species has been appended in the following Table 2. The smaller (10 nm) sized gold (AuNPs) shows grater inhibition on both the Gram positive and Gram negative bacterial species. The larger (40 nm) sized gold (AuNPs) shows lower inhibition compare to smaller AuNPs. This is also lower for K562 cancer cells too.

**Table 2:** Corroborate the MIC (minimum inhibitory concentration) of AuNPs on different bacterial species such as Gram positive and Gram negative

	Bacterial species	MIC (μg mL^−1^)	
		AuNPs-I (∼10 nm)	AuNPs-II (∼40 nm)
Gram positive	*Bacillus subtilis*	11 ± 1.25	10 ± 1.36
	*Bacillus cereus*	11 ± 1.30	10 ± 1.45
	*Staphylococcus aureus*	13 ± 1.11	09 ± 1.76
Gram negative	*Proteus mirabilis*	15 ± 1.97	08 ± 1.66
	*Klebsiella pneumoniae*	17 ± 1.36	08 ± 1.54
	*Escherichia coli*	20 ± 1.53	09 ± 1.29

### 3.5 Cell culture

The synthesized AuNPs of ∼10 nm and ∼40 nm were further used to evaluate its working efficacy through the cell inhibition studies. Initially, K562 (leukaemia) blood cancer cells were cultured in a CO_2_ incubator which is situated in a sterile culture room. However, the 6-well palates were used for the cell culture purpose and each well of the 6-well plate was added with RPMI media which is supplemented with 10% FBS (fetal bovine serum albumin), 100 IU mL^−1^ penicillin, 100 μg mL^−1^ streptomycin and 2 mM l-glutamine. This RPMI medium helps in growing the K562 cells efficiently. They were maintained in a humidified atmosphere with 5% (volume percentage) of CO_2_ (carbon dioxide) at ∼37 °C. The cells were sub-cultured once in every 3 days. The density of around 50 000 cells was taken for cell culture purpose. These cells were stored in liquid nitrogen temperatures (77 K). Finally, the well grown and cultured K562 cells were used for cell inhibition assay with combination of synthesized AuNPs.^11,19,39^

### 3.6 Cell inhibition assay

The cultured K562 cells were used for cell inhibition studies in-association with synthesized ∼10 and 40 nm AuNPs. A series of six (6) wells in a 96-well plate were chosen for cell inhibition assay. However, first well was marked as control or blank well which has been loaded with K562 cells as cultured, which means without any AuNPs. Rest five (5) wells of 96-well plate were poured with an approximate amount of ∼3 × 10^4^ cells per well. These K562 cell containing wells were again added with ∼10 nm sized AuNPs whose concentration is calculated as 0.1 M and each well was added with quantity of 200, 400, 600, 800 and 1000 ng mL^−1^ of AuNPs to each well of 96-well plate.^38–41^ The plate has been kept under slow agitation with 50 RPM for thorough mixing of K562 cells and ∼10 nm sized AuNPs. After thorough mixing, the 96-well plate was incubated in CO_2_ incubator for 36 h treatment between K562 cells and AuNPs with humidified temperature at ∼37 °C. After 36 h treatment followed by the incubation of [(K562 cells)-(∼10 nm sized AuNPs)], the 96-well plate was taken out from CO_2_ incubator and used to measure the cell inhibition (%) followed by the MTT assay.^42,43^ Further, the 96-well plate has been added with purple coloured MTT formazan chemical and plate used to measure the percentage of cell inhibition through the multi-plate reader (Biotek Synergy^4^ model). The Fig. S5† bar graph illustrates the cell inhibition with small sized (∼10 nm) AuNPs. The synthesized ideal sized (∼10 nm) gold nanoparticles were directly incubated with the cultured K562 cells and inhibition measurements performed after 36 h treatment. The cell inhibition (%) for 200, 400, 600, 800 and 1000 ng mL^−1^ of quantities of 0.1 M concentrated AuNPs shows 42, 58, 64, 78 and 88, respectively.^41–45,52^ As the quantity of 0.1 M concentrated AuNPs increases from 200 to 1000 ng mL^−1^, the cell inhibition percentage also increases gradually. This is happened due to insertion and penetration of more number of ideal sized (∼10 nm) AuNPs inside the cell through the permeability of cell membrane.

### 3.7 Cell imaging

**Fig. 8:**
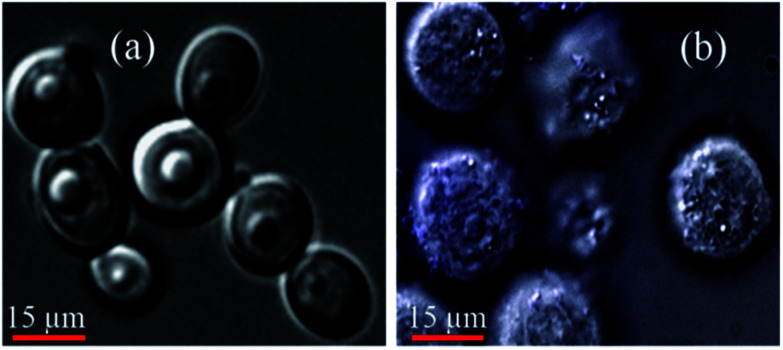
Laser scanning confocal microscopic images of K562 cells illustrate the morphology of cells (a) before treatment which means cells as cultured and (b) cells after treatment with synthesized AuNPs.

The laser scanning confocal microscopic bright field images of K562 cells illustrate the morphology of cells. The cells before (image (a) of Fig. 8) treatment shows clear spherical shaped morphology and tighten cell membrane whereas bright field image of K562 cells (image (b) of Fig. 8) after treatment with synthesized AuNPs shows ruptured and completely disrupted morphology and cell membrane with swelled and fragmented cellular organelles.

The morphology of K562 cells treated with the synthesized ideal sized (∼10 nm) and ∼40 nm AuNPs has been evaluated through the laser scanning confocal microscopy (LSCM) imaging. The 1000 ng mL^−1^ of AuNPs shows significant cell inhibition *i.e.* 88%, which is ever reported inhibition for such ∼10 nm sized AuNPs. Similar method has been followed for the cell inhibition measurements for large sized (∼40 nm) AuNPs. The Fig. S6† bar graph illustrates the cell inhibition (%) with large sized (∼40 nm) AuNPs. The synthesized ∼40 nm sized gold nanoparticles were directly incubated with the cultured K562 cells and inhibition measurements performed after 36 h treatment and found 76% cell inhibition for 1000 ng mL^−1^ quantity of 0.1 M concentrated AuNPs.^44–47,53^

**Fig. 9:**
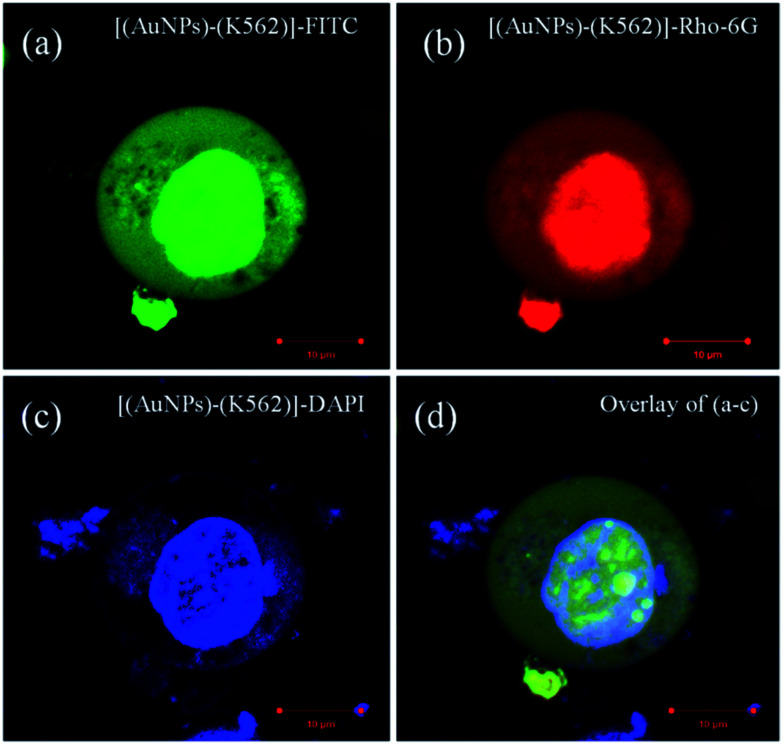
Confocal microscopic images of K562 cells treated with smaller (avg. particle size is 10.4 nm) AuNPs. The cellular morphology for treated cells has been stained with different fluorescent dyes for better understating of size and shape of cells. The image (a) for K562 cell stained with green fluorescent dye FITC (fluorescent isothiocyanate), image (b) corresponds to cell stained with red fluorescent Rho-6G,^48,54^ image (c) corroborates the internal cellular materials stained with blue fluorescent dye DAPI and image (d) corresponds to merge of all the fluorescent images (a–c).

**Fig. 10:**
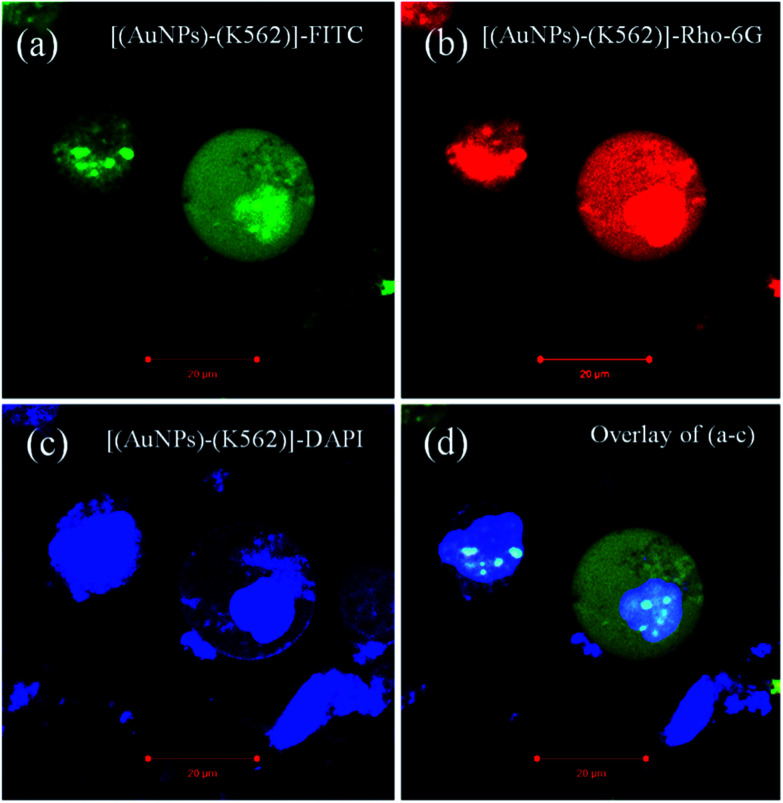
Confocal microscopic images of K562 cells treated with large sized (avg. particle size is 40.2 nm) AuNPs. The cellular morphology for treated cells has been stained with different fluorescent dyes for better understating of size and shape of cells. The image (a) for K562 cell stained with green fluorescent dye FITC (fluorescent isothiocyanate), image (b) corresponds to cell stained with red fluorescent Rho-6G, image (c) corroborates the internal cellular materials stained with blue fluorescent dye DAPI and image (d) corresponds to merge of all the fluorescent images (a–c).

There is around 12% cell inhibition difference for ∼10 nm sized AuNPs to ∼40 nm sized AuNPs and this is happened because of the greater penetration of the ∼10 nm sized AuNPs inside the K562 cells. From this cell inhibition studies, it is confirmed that the larger nanoparticle will not easily enter inside the cell membrane. The ideal sized metal (Au) nanoparticles shows significant permeability followed by the potential cell inhibition efficacy. The Fig. 9a–d corresponds to confocal microscopic images of K562 cells treated with smaller sized (avg. particle size is 10.4 nm) AuNPs. The cellular morphology for treated cells has been stained with different fluorescent dyes for better understating of size and shape of cells. The image (a) is for K562 cell stained with green fluorescent dye FITC (fluorescent isothiocyanate), image (b) corresponds to cell stained with red fluorescent Rho-6G,^48–51,55^ image (c) corroborates the internal cellular materials stained with blue fluorescent dye DAPI and image (d) corresponds to merge of all the fluorescent images (a–c). The Fig. S7 and S8† also corroborate the morphology of K562 cells treated with the ideal sized AuNPs. From these figures, the ruptured morphology of K562 cells has been depicted. Basically, healthy and as cultured K562 cells are spherical in shape without any structural irregularities and distortions. The swelling and shrinkage in cell morphology deliberates the ruptured morphology of cells treated with synthesized AuNPs. Similarly, the Fig. 10a–d and S9a–d† corresponds to confocal microscopic images of K562 cells treated with large sized (avg. particle size is 40.2 nm) AuNPs. From the Fig. S9a–d,† the mitochondria shaped K562 cell can be observed at higher magnifications which is structurally distorted after 36 h treatment with synthesized ∼40 nm sized AuNPs.^11,17,19,43,49,51,56,57^

## 4. Conclusions

Herein, the solvent effect on the synthesis of two different sized (∼10 nm and ∼40 nm) AuNPs has been reported. The affect of polar organic solvent DMF with respect to reducing agent NaBH_4_ gives nanoparticle with an avg. size of 10.4 nm. It has been denoted as the [(DMF)–(NaBH_4_) → (avg. size 10.4 nm)]. Similar synthesis method has been followed with NMPL and Na_3_C_6_H_5_O_7_ and obtained particles with an avg. size of 40.2 nm. It has been denoted as the [(NMPL)–(Na_3_C_6_H_5_O_7_) → (avg. size 40.2 nm)]. The AuNPs size, shape and morphology have been confirmed through the TEM and HRTEM characterizations followed by the SAED patterns. These nanoscale AuNPs were used to evaluate the working efficacy on K562 (leukaemia) blood cancer cells. An *in vitro* cell based studies were performed to examine the potential cell inhibition of synthesized AuNPs and ∼10 nm sized AuNPs gives significant inhibition *i.e.* 88% which is ever reported with such ideal nanosized particles. This ∼10 nm sized AuNPs are recommended for the treatment of breast, lung, and liver cancers and other theranostic applications.

## Conflicts of interest

There are no conflicts to declare.

## Supplementary Material

RA-009-C9RA05484G-s001

## References

[cit1] Amgotha C., Doddapanenib S. J. D. S., Dharmapuric G., Lakavathu M. (2018). Mater. Sci. Eng. C.

[cit2] Kim Y. J., Ryou S., Kim S., Yeom J., Han M. S., Lee K., Seong M. (2012). J. Mater. Chem..

[cit3] Li L., Wanga M., Chen Y., Jiang S. (2012). J. Colloid Interface Sci..

[cit4] Chen T., Xu S., Zhao T., Zhu L., Wei D., Li Y., Zhang H., Zhao C. (2012). ACS Appl. Mater. Interfaces.

[cit5] Dreaden E. C., Alkilany A. M., Huang X., Murphy C. J., El-Sayed M. A. (2012). Chem. Soc. Rev..

[cit6] Kumar A., Boruah B. M., Liang X. (2011). J. Nanomater..

[cit7] Zhang Q., Large N., Wang H. (2014). ACS Appl. Mater. Interfaces.

[cit8] Amgoth C., Dharmapuri G., M Kalle A., Paik P. (2016). Nanotechnology.

[cit9] Singh A., Guleria A., Neogy S., Rath M. C. (2018). Arabian J. Chem..

[cit10] Garcia M., Beecham M. P., Kempe K., Haddleton D. M., Khan A., Marsh A. (2015). Eur. Polym. J..

[cit11] Amgoth C., Lakavathu M., Joshi D. S. D. S. (2018). Bull. Mater. Sci..

[cit12] Lai W., Zhuang J., Que X., Fu L., Tang D. (2014). Biomater. Sci..

[cit13] Baeza A., Guisasola E., Ruiz-Hernandez E., Vallet-Regi M. (2012). Chem. Mater..

[cit14] Perassi E. M., Hrelescu C., Wisnet A., Doblinger M., Scheu C., Jackel F., Coronado E. A., Feldmann J. (2014). ACS Nano.

[cit15] Chaubey N., Sahoo A. K., Chattopadhyay A., Ghosh S. S. (2014). Biomater. Sci..

[cit16] Liang Z., Li X., Xie Y., Liu S. (2014). Biomed. Mater..

[cit17] Gunti R., Dharmapuri G., Doddapaneni S. J., Amgoth C. (2017). Adv. Mater. Lett..

[cit18] Yang C., Bian M., Yang Z. (2014). Biomater. Sci..

[cit19] Amgoth C., Joshi S. (2017). Mater. Res. Express.

[cit20] Koch A. H. R., Leveque G., Harms S., Jaskiewicz K., Bernhardt M., Henkel A., Sonnichsen C., Landfester K., Fytas G. (2014). Nano Lett..

[cit21] Talom R. M., Fuks G., Mingotaud C., Gineste S., Gauffre F. (2012). J. Colloid Interface Sci..

[cit22] Amgotha C., Dharmapuri G. (2016). Mater. Today: Proc..

[cit23] Ganguly A., Trovato O., Duraisamy S., Benson J., Han Y., Satriano C., Papakonstantinou P. (2019). J. Phys. Chem. C.

[cit24] Gupta R., Rai B. (2017). Sci. Rep..

[cit25] Wu X., Tian Y., Yu M., Hanb J., Han S. (2014). Biomater. Sci..

[cit26] Li W., Zhu X., Wang J., Liang R., Li J., Liu S., Tu G., Zhu J. (2014). J. Colloid Interface Sci..

[cit27] Hove J., Schijven L. M. I., Wang J., Velders A. H. (2018). Chem. Commun..

[cit28] Liu L., Zhang X., Chaudhuri J. (2014). Mater. Res. Express.

[cit29] La Y., Park C., Shin T. J., Joo S. H., Kang S., Kim K. T. (2014). Nat. Chem..

[cit30] Balasubramanian R., Xu J., Kim B., Sadtler B., Wei A. (2001). J. Dispersion Sci. Technol..

[cit31] Zhang Q., Large N., Nordlander P., Wang H. (2014). J. Phys. Chem. Lett..

[cit32] Martin M. N., Basham J. I., Chando P., Eah S. (2010). Langmuir.

[cit33] Shervani Z., Yamamoto Y. (2011). Mater. Lett..

[cit34] Singh A., Guleria A., Kunwar A., Neogy S., Rath M. C. (2019). Mater. Res. Express.

[cit35] Chu Z., Zhang S., Yin C., Lin G., Li Q. (2014). Biomater. Sci..

[cit36] Letchford K., Burt H. (2007). Eur. J. Pharm. Biopharm..

[cit37] Chen H., Chi X., Li B., Zhang M., Ma Y., Achilefud S., Gu Y. (2014). Biomater. Sci..

[cit38] Craig D. Q. M. (2002). Int. J. Pharm..

[cit39] Shen S., Chen X., Zhang X., Miao J., Zhao B. (2015). J. Mater. Chem. B.

[cit40] Bradburne C. E., Delehanty J. B., Gemmill K. B., Mei B. C., Mattoussi H., Susumu K., Blanco-Canosa J. B., Dawson P. E., Medintz I. L. (2013). Bioconjugate Chem..

[cit41] Fan J., Zeng F., Wu S., Wang X. (2012). Biomacromolecules.

[cit42] Yang Y., Wang W., Chen T., Chen Z. (2014). ACS Appl. Mater. Interfaces.

[cit43] Hu M., Chen M., Li G., Pang Y., Wang D., Wu J., Qiu F., Zhu X., Sun J. (2012). Biomacromolecules.

[cit44] Clear K. J., Harmatys K. M., Rice D. R., Wolter W. R., Suckow M. A., Wang Y., Rusckowski M., Smith B. D. (2015). Bioconjugate Chem..

[cit45] Singh A., Kunwar A., Rath M. C. (2018). J. Nanosci. Nanotechnol..

[cit46] Singh A., Guleria A., Kunwar A., Neogy S., Rath M. C. (2017). Mater. Chem. Phys..

[cit47] Shukla S., Dickmeis C., Nagarajan A. S., Fischer R., Commandeur U., Steinmetz N. F. (2014). Biomater. Sci..

[cit48] Abinayasri P., Nageshwari M., Meenarathi B., Anbanbarasan R. (2017). Bull. Mater. Sci..

[cit49] Feng W., Zhou X., He C., Qiu K., Nie W., Chen L., Wang H., Mo X., Zhang Y. (2013). J. Mater. Chem. B.

[cit50] Jin Kim Y., Ryou S., Kim S., Yeom J., Han M., Lee K., Seong M. (2012). J. Mater. Chem..

[cit51] Zhang Q., Zhu J., Song L., Zhang J., Kong D., Zhao Y., Wang Z. (2013). J. Mater. Chem. B.

[cit52] Mu Q., Jiang G., Chen L., Zhou H., Fourches D., Tropsha A., Yan B. (2014). Chem. Rev..

[cit53] Huang Y., Liu Q., Wang Y., He N., Zhao R., Choo J., Chen L. (2019). Nanoscale.

[cit54] Yu Q., Wang Y., Mei R., Yin Y., You J., Chen L. (2019). Anal. Chem..

[cit55] Mei R., Wang Y., Liu W., Chen L. (2019). ACS Appl. Mater. Interfaces.

[cit56] Zhang W., Wang Y., Sun X., Wang W., Chen L. (2014). Nanoscale.

[cit57] Park H., Lee S., Chen L., Lee E. K., Shin S. Y., Lee Y. H., Son S. W., Oh C. H., Song J. M., Kang S. H., Choo J. (2009). Phys. Chem. Chem. Phys..

