# Supplementary material for: Solvent assisted size effect on AuNPs and significant inhibition on K562 cells
Source: RSC Adv. 2019 Oct 22;9(58):33931–40. doi: 10.1039/c9ra05484g (PMC9073664; doi:10.1039/c9ra05484g)
Supplement: RA-009-C9RA05484G-s001 [file RA-009-C9RA05484G-s001.pdf]

Electronic Supplementary Material (ESI) for RSC Advances.
This journal is © The Royal Society of Chemistry 2019

Supporting Information

Solvent Assisted Size Effect on AuNPs and Significant Inhibition on K562 Cells

Chander Amgoth[a,1*], Avinash Singh[a,2], Sujata Yumnam[a,3], Priyanka Mangla[a,4], R Karthik[b], Tang Guping[c], Murali Banavoth[d,*]

[a]*Department of Science and Humanities, MLR Institute of Technology, Hyderabad-500043,TS, India*
[b]*Department of Electronics and Communication Engineering, MLR Institute of Technology, Hyderabad-500043, TS, India*
[c]*School of Chemistry, Zhejiang University, Hangzhou-310028, China.*
[d]*School of Chemistry, University of Hyderabad, Hyderabad-500046,TS, India*

*Corresponding author: E-mail: chander@uohyd.ac.in

Characterizations Techniques

Following list of instruments was used to characterize the (Gly) and ($MPSiO_2NPs$) based composite.

(1) Digital electronic microbalance (Acculab), (2) Homogenizer (REMI, RQ 127A), (3) Micro centrifuge (Genei) for separation, (4) Micro-pipette (Accupipet), (5) CO_2 incubator (Equitron, Medica Inst) for cell culture and cell inhibition studies (5% volume of CO_2 at ~37 °C, (6) Sonicator (Ultrasonic cleaner, Enertech Electronics), (7) Multiplate Reader for MTT assay (cell inhibition studies) BioTeck Synergy H^4 model, (8) CM (confocal microscope) for fluorescence imaging experiments using Zeiss LSM 700 model microscope, (9) EDAX (Energy dispersive spectroscopy) to analyze the elemental composition present in [(Gly)-(SiO_2NPs)], (10) SEM (Scanning electron microscope), (11) TEM (Transmission electron microscope), (12) TGA (Thermogravimetric analysis), (13) DSC (Differential scanning calorimetry), (14) XRD (X-Ray diffraction).

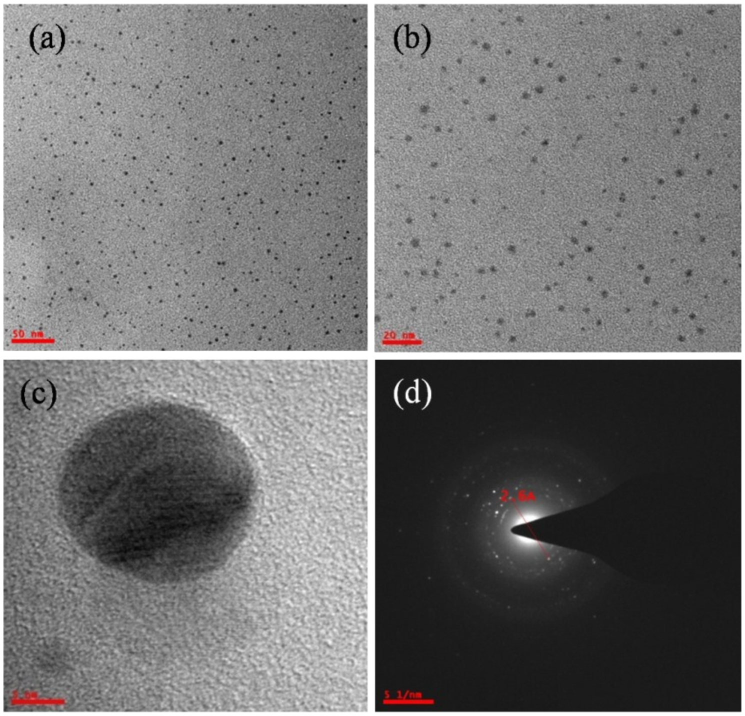

Fig. S1 TEM micrographs illustrate the smaller gold nanoparticles (Avg. size 10.4 nm) and images (a-c) are acquired from lower to higher magnifications and corroborate the dispersion and distribution over the surface of the TEM grid. The image (d) corresponds to SAED for AuNPs crystalline metal nanoparticles and corroborates the de-spacing value as 2.6 ◦A.

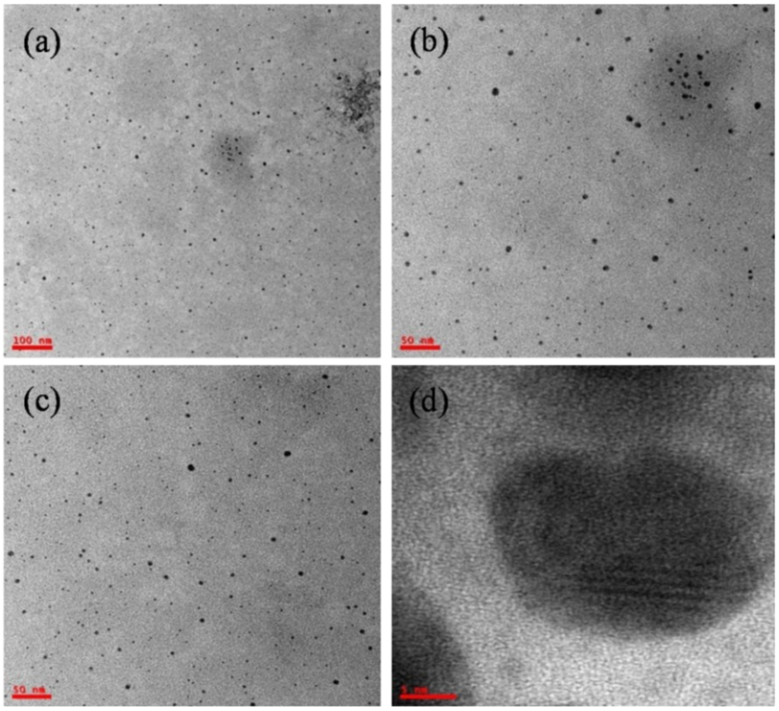

Fig. S2 TEM micrographs illustrate the smaller gold nanoparticles (Avg. size 10.4 nm) and images (a-d) are acquired from lower to higher magnifications and corroborate the dispersion and distribution over the surface of the TEM grid.

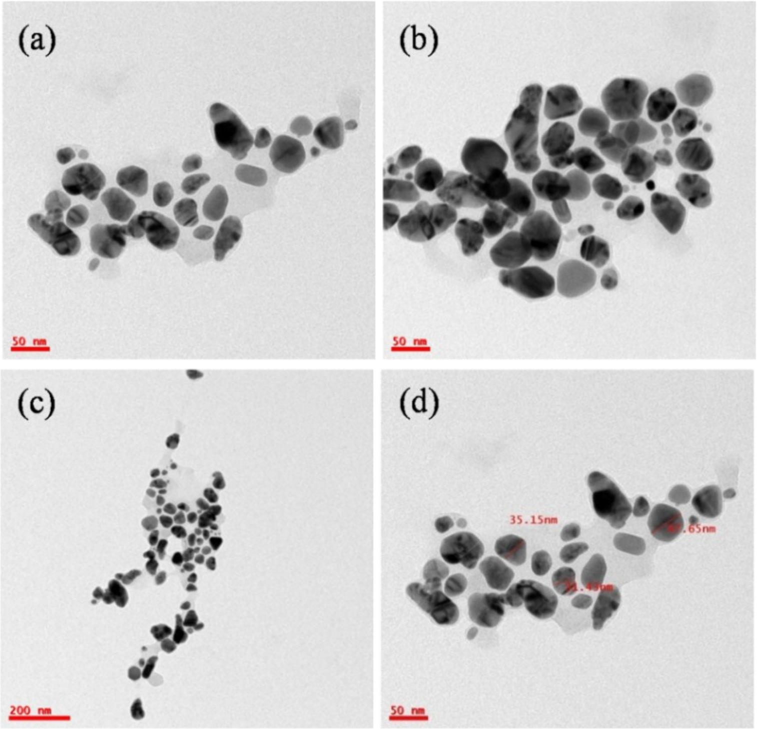

Fig. S3 TEM micrographs for synthesized AuNPs for larger gold nanoparticles (Avg. particle size is 40.2 nm). The images (a-d) are acquired from lower to higher magnifications. The image (d) represent the size of the AuNPs are red marked with respect to particle diameter.

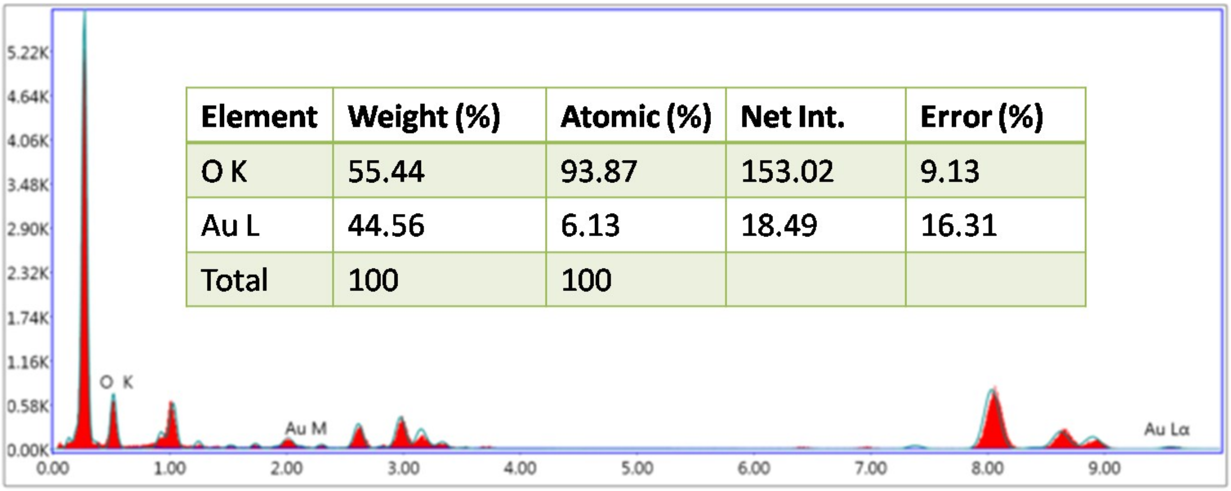

Element	Weight (%)	Atomic (%)	Net Int.	Error (%)
O K	55.44	93.87	153.02	9.13
Au L	44.56	6.13	18.49	16.31
Total	100	100		

Fig. S4 EDAX (Energy dispersive X-ray spectrometer) for synthesized AuNPs. The elemental composition of oxygen (O) is because of impurities present in the sample. The in-set table shows the atomic and weight percentages of Au element.

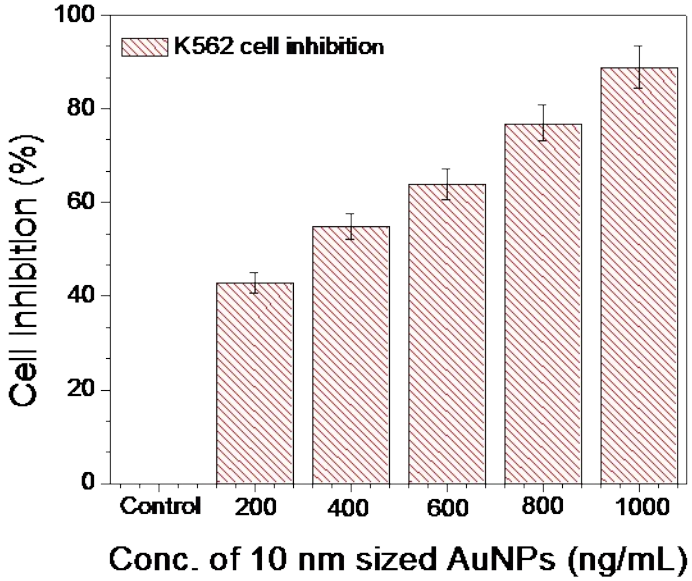

Fig. S5 Bar graph illustrates the cell inhibition with small sized (10 nm) AuNPs. The synthesized 10 nm sized gold nanoparticles were directly incubated with the cultured K562 cells and inhibition measurements performed after 36 h treatment and found 88% inhibition for 1000 ng/mL of AuNPs.

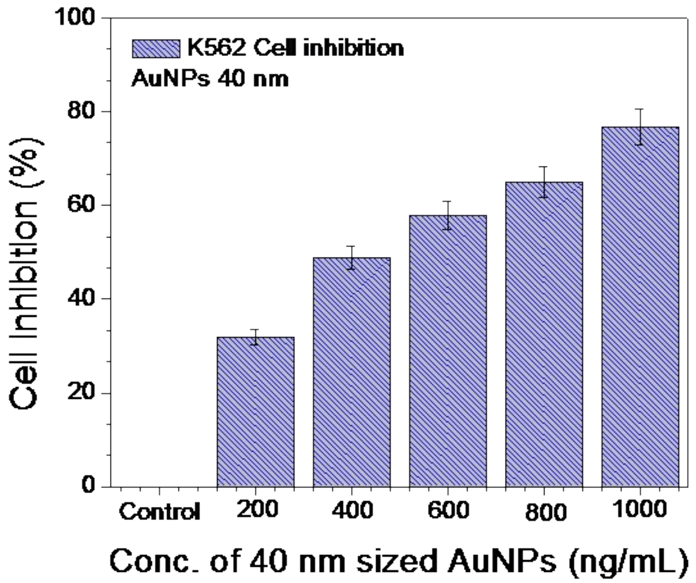

Fig. S6 Bar graph illustrates the cell inhibition with large sized (40 nm) AuNPs. The synthesized 40 nm sized gold nanoparticles were directly incubated with the cultured K562 cells and inhibition measurements performed after 36 h treatment and found 76% inhibition for 1000 ng/mL concentration of AuNPs.

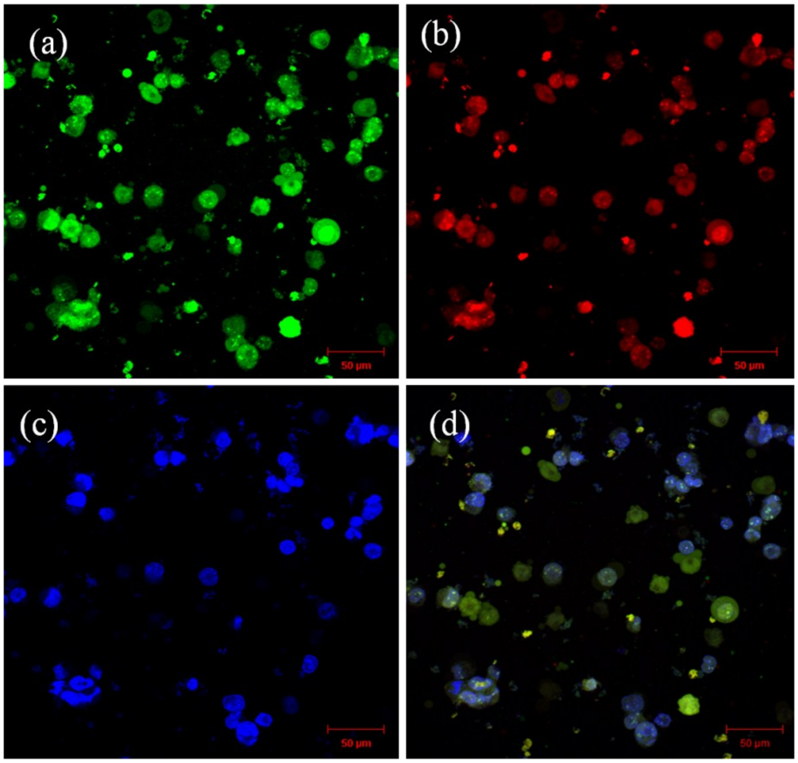

Fig. S7 Confocal microscopic images of K562 cells acquired at lower magnifications. These cells were treated with small (10 nm) sized AuNPs. Scale bar 50 µm.

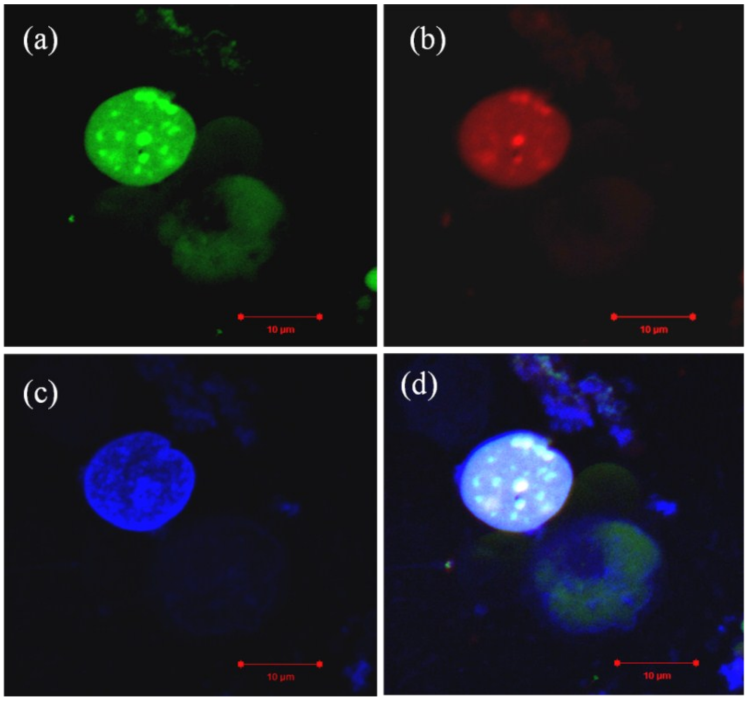

Fig. S8 Confocal microscopic images of K562 cells acquired at higher magnification. These cell were treated with small (10 nm) sized AuNPs. The scale bar is 10 µm.

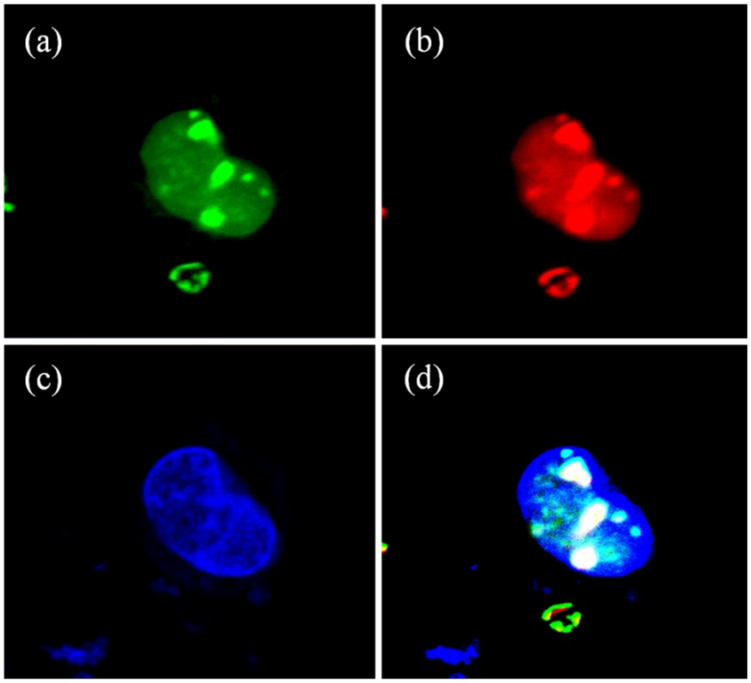

Fig. S9 Confocal microscopic images of K562 cells acquired at higher magnifications. The cells were treated with large (40 nm) sized AuNPs. The cellular morphology for treated cells has been stained with different fluorescent dyes for better understating of size and shape of cells. The image (a) for K562 cell stained with green fluorescent dye FITC (fluorescent isothiocyanate), image (b) corresponds to cell stained with red fluorescent Rho-6G, image (c) corroborates the internal cellular materials stained with blue fluorescent dye DAPI and image (d) corresponds to merge of all the fluorescent images (a-c).
